# A High Throughput Isolation Method for Phosphate-Accumulating Organisms

**DOI:** 10.1038/s41598-019-53429-2

**Published:** 2019-12-02

**Authors:** Ajeeta Anand, Hideki Aoyagi

**Affiliations:** 1 Institute of Life Sciences and Bioengineering, Graduate School of Life and Environmental Sciences, University of Tsukuba, Tsukuba, Ibaraki 305-8572 Japan; 2 Faculty of Life and Environmental Sciences, University of Tsukuba, Tsukuba, Ibaraki 305-8572 Japan

**Keywords:** Applied microbiology, High-throughput screening, Industrial microbiology

## Abstract

Hyperphosphatemia is a secondary issue associated with chronic kidney disorder. Use of phosphate binders and dialysis are the treatments for hyperphosphatemia, albeit with harmful side effects and high cost, respectively. A safer and healthier approach is attempted to administer phosphate-accumulating organisms (PAOs) from probiotics to prevent hyperphosphatemia. However, screening and isolation of PAOs are limited by inefficient enrichment of relevant metabolism and contamination. Therefore, we devised a novel strategy to isolate elite PAOs from *Lactobacillus casei* JCM 1134 and *Bifidobacterium adolescentis* JCM 1275 (previously reported PAOs). PAOs were first enriched for phosphate uptake and incubated in low-pH phosphate-free media to dormant non-PAOs, and then purified using Percoll density gradient centrifugation. Subsequently, elite PAOs were isolated from centrifuged pellet on a toluidine blue O-supplemented agar-based media. Using this technique, elite PAOs could not only be isolated, but also semi-quantitatively scored for their phosphate accumulation capabilities. Additionally, these scores correlated well with their accumulated phosphate values. The elite PAOs isolated from *L. casei* and *B. adolescentis* showed 0.81 and 0.70 [mg-phosphate/mg-dry cell], respectively (23- and 4.34-fold increase, respectively). Thus, our method can be used to successfully isolate elite PAOs, which might be of use to prevent hyperphosphatemia at early stages.

## Introduction

Pervasive phosphate-rich food habits and work pressure co-exist with chronic kidney disorder (CKD), one of the major causes of hyperphosphatemia due to deficiencies in kidney function^1^. Hyperphosphatemia is followed by a cascade of several other diseases, such as hypocalcaemia, renal bone disorders, and abnormal calcification of vasculatures^1^. Generally, serum phosphate levels above 6.5 mg/dL are linked to high risk of mortality^2^. According to the National Kidney Foundation-Kidney Disease Quality Outcome Initiative, CKD patients on dialysis should maintain serum phosphate levels between 3.5 to 5.5 mg/dL (National kidney foundation, accessed on 1^st^ feb, 2018)^3^. Currently, CKD-associated hyperphosphatemia is treated by reduction of dietary phosphate and phosphate chelation by chemotherapy and dialysis^1^. Protein-restricted diets are also prescribed; however, these may lead to protein deficiency and poor quality of life that further worsen by phosphate-restricted diet and dialysis with frequent, costly, and long schedules^4^. Moreover, chemotherapy using phosphate binders such as calcium carbonate, lanthanum carbonate, ferric citrate, and Sevelamer can elicit hypercalcemia, deposition of lanthanum in the bone, and gastric issues such as constipation and vomiting, respectively^5^. Indeed, all these treatments are prescribed typically at advanced stages of CKD when kidney damage is almost irreversible and the maintenance of normal serum phosphate levels becomes a lifelong challenge with associated side effects.

The potential of applying phosphate-accumulating organisms (PAOs) to prevent hyperphosphatemia has not yet been completely investigated. PAOs have been identified, particularly in wastewater; for example, *Acinetobacter* spp. was isolated by enrichment on acetate agar and identified as an efficient wastewater PAO^6^. Similarly, yeast cultures enriched in phosphate-rich media have been used to remove phosphate load from waste streams^7^. Optimisation of enrichment parameters for PAO isolation from waste water, such as supplementation with citrate, Mg^2+^, Ca^2+^, and phosphate led to the identification of *Serratia marcescens* as a potent PAO^8^. The reported methods for PAO isolation are neither specific for the enrichment of the relevant metabolism by optimizing significant parameters nor strategic to remove non-PAOs completely and efficiently. Thus, contamination of non-PAOs and isolation of PAOs with higher potential are still challenging issues that need to be addressed. Although, wastewater PAOs cannot be administered as therapy for humans, PAOs among edible intestinal bacteria (probiotics) may potentially prevent hyperphosphatemia^9^. Probiotics as PAOs is a trustworthy approach, as probiotics are already in widely use as functional foods, do not require medical prescription, and are known to confer health benefits. Therefore, administration of probiotic PAOs as functional food may prevent hyperphosphatemia while maintaining the quality of life.

**Figure 1:**
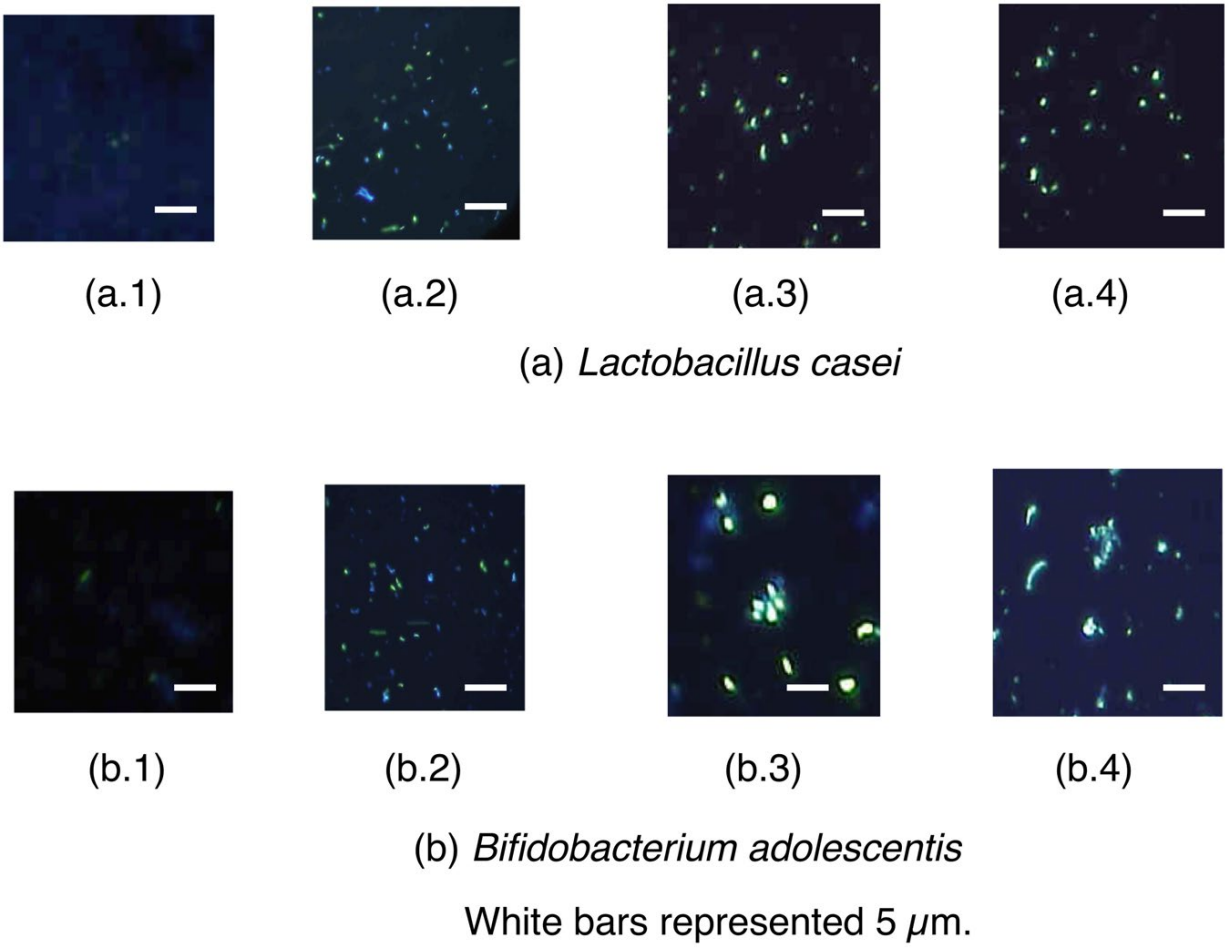
DAPI micrographs of (**a**) *L. casei* JCM 1134 and (**b**) *B. adolescentis* JCM 1275. **a.1 and b.1**: Cells after 18 h in phosphate accumulation media. **a.2 and b.2**: Cells after 2 h in phosphate-free media at pH 3.5 and 4.0, respectively. **a.3 and b.3**: Cells after Percoll density gradient centrifugation. **a.4 and b.4**: L11 and B8 isolates after 18 h in phosphate accumulation media.

Previously, we identified the probiotics *Lactobacillus casei* JCM 1134 and *Bifidobacterium adolescentis* JCM 1275 as potential PAOs^9^, although these strains were contaminated with non-PAOs (blue cells in Fig. 1 a.1 and b.1), which decreased the total efficiency of phosphate accumulation. Accordingly, to facilitate the isolation of elite PAO strains from *L. casei* JCM 1134 and *B. adolescentis* JCM 1275, new strategies were designed to enrich relevant metabolic characteristics and remove undesired non-PAOs. Toward this objective, we developed a novel and comprehensive method that involved the enrichment of phosphate accumulation metabolism in phosphate-accumulation media (optimized), subsequent inactivation of non-PAOs in a low-pH phosphate-free media, and separation via centrifugation on a Percoll density gradient. A novel semiquantitative assay was formulated to isolate elite PAO strains and score their potential of phosphate accumulation based on the size of the coloured colony on agar media supplemented with toluidine blue O (TBO), a chromogen that forms a coloured complex with phosphate.

## Results

### Phosphate accumulation

*L. casei* JCM 1134 and *B. adolescentis* JCM 1275 showed 0.049 and 0.178 [mg-phosphate/mg-dry cell], respectively after 36 h in phosphate-accumulation medium **(**Table 1**)**. However, intracellular phosphate increased exponentially during the initial 18 h (0.035 and 0.160 [mg-phosphate/mg-dry cell] for *L. casei* JCM 1134 and *B. adolescentis* JCM 1275, respectively), and a gradual increment in phosphate content was observed later, as measured using the ascorbic acid method **(**Table 1**)** and supported by staining with 4′, 6-diamidino-2-phenylindole (DAPI) dye **(**Fig. 1 a.1 and b.1**)**. Therefore, 18 h of incubation in phosphate-accumulation medium was selected for further studies.

**Table 1:** Growth characteristics and phosphate content of cells in phosphate accumulation media.

Time (h)	*L. casei* JCM 1134				*B. adolescentis* JCM1275			
	OD at 680 nm	pH	Phosphate (mg/mg-dry cell)	CFU/mL (×10^2^)	OD at 680 nm	pH	Phosphate (mg/mg-dry cell)	CFU/mL (×10^2^)
6	0.31 ± 0.01	6.0 ± 0.2	0.012 ± 0.01	4.10 ± 0.2	0.34 ± 0.02	6.1 ± 0.2	0.102 ± 0.02	5.60 ± 0.2
12	0.28 ± 0.02	5.9 ± 0.1	0.029 ± 0.01	3.80 ± 0.4	0.32 ± 0.03	5.9 ± 0.1	0.132 ± 0.03	5.20 ± 0.2
18	0.26 ± 0.01	5.9 ± 0.3	0.035 ± 0.02	2.83 ± 0.3	0.30 ± 0.04	5.9 ± 0.6	0.160 ± 0.01	3.20 ± 0.1
24	0.26 ± 0.01	5.8 ± 0.2	0.040 ± 0.03	2.79 ± 0.2	0.28 ± 0.01	5.9 ± 0.2	0.168 ± 0.04	2.78 ± 0.3
30	0.25 ± 0.01	5.8 ± 0.7	0.042 ± 0.01	2.64 ± 0.3	0.28 ± 0.03	5.8 ± 0.3	0.172 ± 0.02	2.67 ± 0.1
36	0.23 ± 0.03	5.8 ± 0.5	0.049 ± 0.02	2.24 ± 0.7	0.24 ± 0.01	5.8 ± 0.2	0.178 ± 0.01	2.45 ± 0.5
42	0.19 ± 0.01	5.8 ± 0.8	0.043 ± 0.04	1.45 ± 0.3	0.20 ± 0.06	5.7 ± 0.8	0.174 ± 0.03	2.03 ± 0.6
48	0.22 ± 0.01	5.7 ± 0.2	0.043 ± 0.05	0.56 ± 0.1	0.16 ± 0.07	5.7 ± 0.2	0.163 ± 0.05	1.09 ± 0.6
54	0.12 ± 0.02	5.7 ± 0.1	0.032 ± 0.02	0.50 ± 0.5	0.16 ± 0.03	5.7 ± 0.1	0.152 ± 0.06	0.20 ± 0.2

Data are presented as means ± standard errors of mean, n = 3 and *p* value < 0.05 was considered significant.

However, the cultures contained strains with diverse range of phosphate accumulating abilities, as low potential PAOs as well as non-PAOs were observed in DAPI micrographs (cells stained with light yellow and blue, respectively in Fig. 1 a.1 and b.1). Hence, a suitable isolation method was designed to enrich PAOs and remove non-PAOs, following which a new semiquantitative method was used to compare phosphate accumulation capabilities of the PAOs and thereby select elite PAO strains.

### Isolation of elite PAOs

#### First step: selection in low pH

**Figure 2:**
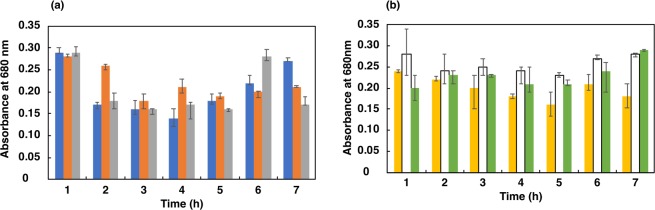
Growth of *L. casei* JCM 1134 at (**a**) pH 3.5 (), 4.0 (), and 5.0 (), and at (**b**) pH 6.0 (), 7.0 (□), and 8.0 (). Standard errors are expressed as error bars and all the values are significantly different at *p* value < 0.05, except for pH 4.0 at 1 h and 2 h.

**Figure 3:**
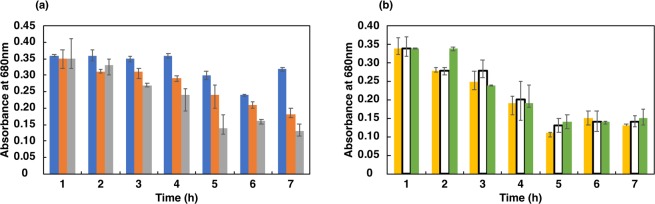
Growth of *B. adolescentis* JCM 1275 at (**a**) pH 3.5 (), 4.0 (), and 5.0 (), and at (**b**) pH 6.0 (), 7.0 (□), and 8.0 (). Standard errors are expressed as error bars and all the values are significantly different at *p* value < 0.05, except for pH 3.5 at 1 h and 2 h.

**Figure 4:**
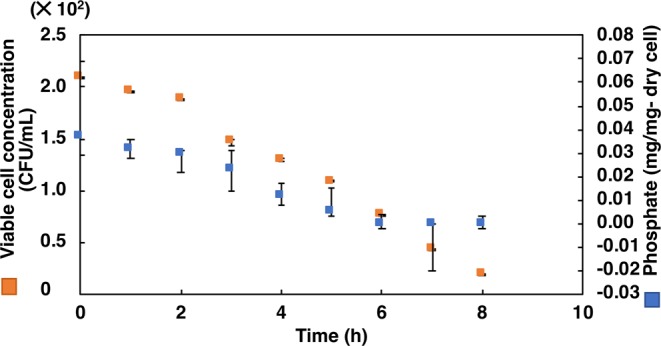
*L. casei* JCM 1134 in phosphate accumulation media (phosphate-free) pH 4.0. Standard errors are expressed as error bars and all the values are significantly different at *p* value < 0.05, except for the values from 0 h to 2 h.

**Figure 5:**
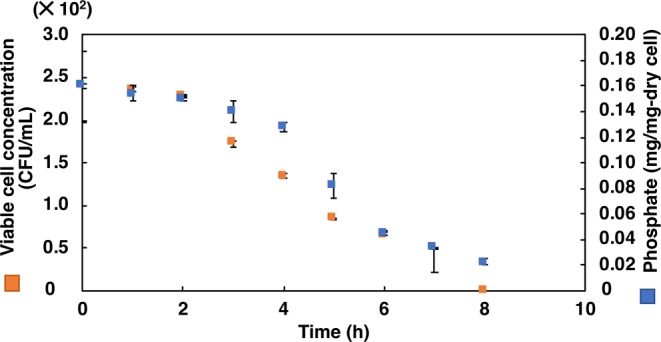
*B. adolescentis* JCM 1275 in phosphate accumulation media (phosphate-free) pH 4.0. Standard errors are expressed as error bars and all the values are significantly different at *p* value < 0.05, except for the values from 0 h to 2 h.

Incubation of cultures at extremely low pH showed that the optical density at 680 nm decreased drastically with time (Figs. 2 and 3), except at pH 4.0 for *L. casei* JCM 1134 and pH 3.5 for *B. adolescentis* JCM 1275 in 2 h of incubation. However, viability (in CFU/mL) decreased at these pH values after 2 h of incubation, suggesting that the cells had become dormant or dead (Figs. 4 and 5). This result corroborated the maintenance of phosphate content for up to 2 h **(**Figs. 4 and 5**)**. Increased optical density (at 680 nm) at later hours of incubation suggested that acid-resistant *L. casei* JCM 1134 and *B. adolescentis* JCM 1275 metabolised the debris of dead cells as carbon and nitrogen sources **(**Figs. 2 and 3**)**. No significant loss of cells was observed for up to 2 h at pH 4.0 and 3.5 for *L. casei* JCM 1134 and *B. adolescentis* JCM 1275, respectively **(**Figs. 2 and 3).

To verify the increase in phosphate accumulation efficiency after selection at low pH for 2 h, active cultures were re-introduced into phosphate-accumulation media and incubated for 18 h. Subsequently, DAPI staining showed a significantly higher population of PAOs than non-PAOs **(**Fig. 1 a.2 and b.2**)**, with a phosphate content of 0.598 and 0.432 [mg-phosphate/mg-dry cell] for *L. casei* JCM 1134 and *B. adolescentis* JCM 1275, respectively. Therefore, selection in low-pH phosphate-free media enhanced the phosphate content approximately by 17- and 2.7-fold compared with the initial cultures and that improved their phosphate accumulation capabilities remarkably. This low pH incubation strategy causes dormancy or death of non-PAOs, allowing PAOs to become dominant over non-PAOs.

#### Second step: percoll centrifugation

After selection in low pH, we attempted to separate the PAOs from the acid resistant non-PAOs. Percoll density gradient centrifugation was performed at suitable NaCl osmolarity, as PAOs are denser than non-PAOs due to the accumulated polyphosphate granules. Under optimal concentrations of 70% and 95% v/v Percoll, phosphate-accumulating *L. casei* JCM 1134 and *B. adolescentis* JCM 1275, respectively, accounted for the majority of cells in the denser part after centrifugation (Fig. 1 a.3 and b.3**)**. The separated cells from denser part were then grown in phosphate-accumulation medium for 18 h at 37 °C and were found to contain 0.671 and 0.582 [mg-phosphate/mg-dry cell] for *L. casei* JCM 1134 and *B. adolescentis* JCM 1275, respectively, indicating that Percoll density gradient centrifugation in conjunction with first step,  strongly enhanced not only the phosphate content approximately by 19.2-fold and 3.64-fold, respectively but also their phosphate accumulation capabilities.

#### Third step and data processing: TBO agar assay

PAOs purified from *L. casei* JCM 1134 and *B. adolescentis* JCM 1275 were highly diverse in terms of phosphate uptake. Hence, further screening was performed on phosphate-rich and phosphate-deficient agar plates to isolate elite PAO strains. Media were supplemented with TBO, which forms a coloured complex with phosphate and is metabolised successfully as colonies grow. At suitable dilution, the colonies can be scored for their phosphate accumulation capabilities in terms of the stained region [Eq. (1)], which correlates with the concentration of stored intracellular phosphate; colonies on phosphate-deficient media were used as reference. Fifteen colonies of *L. casei* JCM 1134 and ten of *B. adolescentis* JCM 1275 were considered potential PAOs (Table 2).

**Table 2:** Phosphate contents of 15 isolates of *L. casei* JCM 1134 and 10 isolates of *B. adolescentis* JCM 1275.

*L. casei* JCM 1134 isolates	Phosphate (mg/mg-dry cell)	Plate assay	*B. adolescentis* JCM 1275 isolates	Phosphate (mg/mg-dry cell)	Plate assay
L1	0.60 ± 0.01	0.013 ± 0.002	B1	0.67 ± 0.03	0.030 ± 0.002
L2	0.63 ± 0.02	0.024 ± 0.003	B2	0.63 ± 0.04	0.022 ± 0.006
L3	0.69 ± 0.01	0.034 ± 0.006	B3	0.59 ± 0.02	0.010 ± 0.008
L4	0.76 ± 0.03	0.043 ± 0.008	B4	0.60 ± 0.03	0.012 ± 0.002
L5	0.71 ± 0.02	0.037 ± 0.006	B5	0.65 ± 0.02	0.026 ± 0.006
L6	0.64 ± 0.02	0.026 ± 0.003	B6	0.62 ± 0.01	0.019 ± 0.001
L7	0.70 ± 0.01	0.034 ± 0.002	B7	0.66 ± 0.01	0.029 ± 0.006
L8	0.74 ± 0.03	0.040 ± 0.004	B8	0.70 ± 0.07	0.036 ± 0.002
L9	0.77 ± 0.03	0.046 ± 0.008	B9	0.57 ± 0.02	0.002 ± 0.001
L10	0.67 ± 0.01	0.032 ± 0.002	B10	0.59 ± 0.01	0.009 ± 0.005
L11	0.81 ± 0.04	0.052 ± 0.002			
L12	0.76 ± 0.05	0.045 ± 0.007			
L13	0.77 ± 0.02	0.047 ± 0.002			
L14	0.70 ± 0.01	0.037 ± 0.006			
L15	0.72 ± 0.03	0.040 ± 0.008			

Data are presented as means ± standard errors of mean, n = 3, and *p* value < 0.05 was considered significant.

### Efficiency of elite PAOs using semi-quantitative and quantitative methods

**Figure 6:**
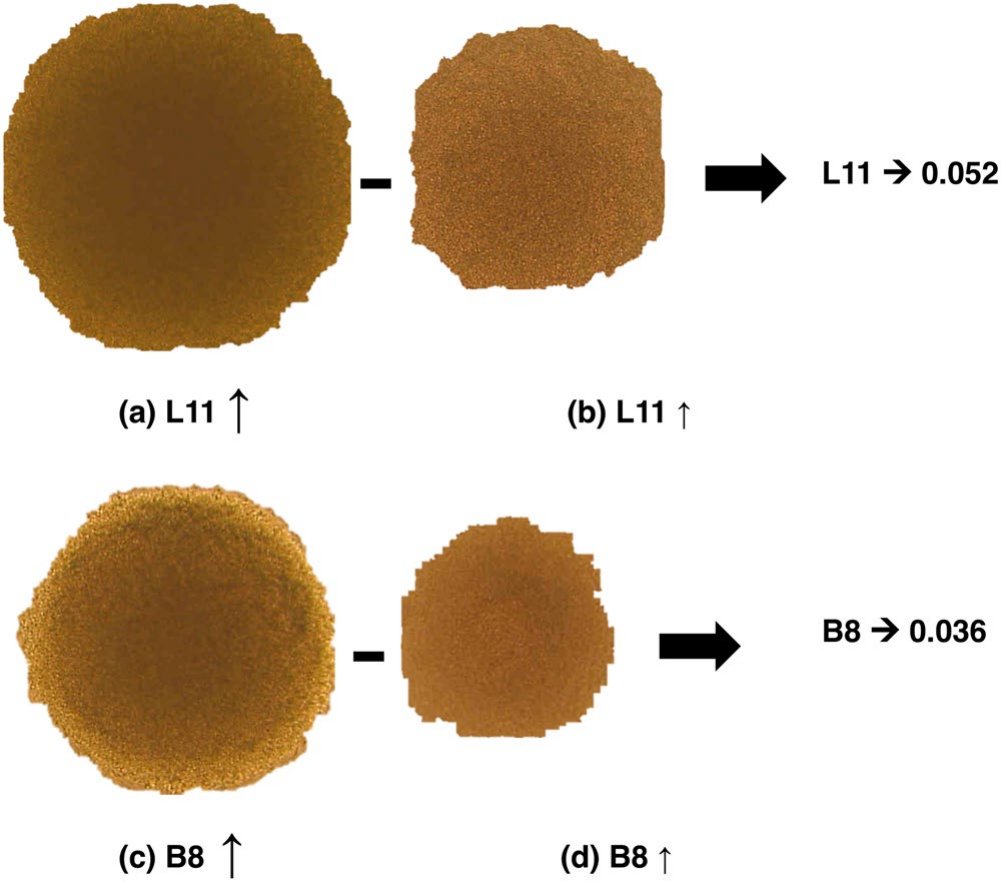
Semi-quantitative scoring of phosphate accumulation in isolate L11 from *L. casei* JCM 1134 and isolate B8 from *B. adolescentis* JCM 1275, using Microsoft PowerPoint 2016. Colonies were grown in (**a**) phosphate-rich (2.2% w/v) MRS medium, (**b**) phosphate-deficient (0.2% w/v) MRS medium, (**c**) phosphate-rich (2.3% w/v) B medium, and (**d**) phosphate-deficient (0.3% w/v) B medium. The length of the arrow represents the comparative size of the stained area.

The isolated elite PAOs were grown in phosphate-accumulation medium for 18 h at 37 °C and assayed for intracellular phosphate using the ascorbic acid method (Table 2). The obtained quantitative results of phosphate accumulation were found to correlate well with the semi-quantitative scores generated by TBO assay. Isolates L11 from *L. casei* JCM 1134 and B8 from *B. adolescentis* JCM 1275 were the most efficient phosphate accumulators or elite PAOs and scored 0.052 and 0.036 in the semi-quantitative assay, respectively **(**Fig. 6**)**. L11 and B8 strains showed 0.81 and 0.7 [mg-phosphate/mg-dry cell], respectively, which corresponded to a significant 23- and 4.34-fold increase in phosphate content compared with the initial cultures; this was also confirmed by their DAPI micrographs (Fig. 1 a.4 and b.4**)**. A pictorial representation of the enhancement in phosphate accumulation capabilities of *L. casei* JCM 1134 and *B. adolescentis* JCM 1275 during isolation of PAOs in stepwise manner is shown in Supplementary Fig. S1.

## Discussion

To prevent hyperphosphatemia by a safer and healthier microbial approach, PAOs have to be isolated as pure cultures with efficient phosphate accumulation capabilities^10^. Toward this objective, cultures were incubated in phosphate-accumulation media, which induced phosphate accumulation and enriched PAOs (Table 1 and Fig. 1). Importantly, enrichment of PAOs using this medium was not species-specific, as relevant metabolic pathways are directly induced by the salts provided. As per literature, phosphate-deficient media enhanced the survival of PAOs over other strains^11^. To select against non-PAOs, the cultures were incubated in phosphate-free medium at pH 3.5, 4.0, 5.0, 6.0, 7.0, and 8.0; pH 3.5 was used as the lower limit because Mg^2+^ was reported to be released from target cells at pH < 3.5, thereby destabilising polyphosphates^12^.

To maintain pH homeostasis at pH 5.0 to 7.0, bacterial cells incorporate phosphate ions from the positively charged components of the cell membrane via active transport. However, cells were reported to express the F_1_F_0_ ATPase more abundantly at pH 8.0 or above, and expel hydronium ions to maintain pH homeostasis^13^. However, in this study, these survival strategies were ineffective in the high pH media, as cell death was eventually observed and ultimately resulted in the loss of both PAOs and non-PAOs (Figs. 2 and 3).

No significant loss in optical density was observed up to 2 h at pH 4.0 and 3.5 for *L. casei* JCM 1134 and *B. adolescentis* JCM 1275, respectively (Figs. 2 and 3). To survive at these low pH values, symport and F_1_F_0_ATPase expression were reported to be ineffective to maintain pH homeostasis^12^. Indeed, phosphate-rich PAOs adapted at low pH by expelling phosphate as a base to achieve pH balance (as phosphate was detected in culture supernatants) and other essential metabolic functions. In contrast, non-PAOs became dormant or died, likely because they are incapable of survival at low pH by expelling phosphate or any other means, and thereby cannot maintain cellular homeostasis. Reports showed that PAOs utilise the stored phosphate as an energy source during adverse conditions. Mino *et al*. (1985) and Fuhs and Chen (1975) detected low- and high-molecular weight polyphosphates in PAOs, and suggested that the latter may provide energy under anaerobic conditions^14^. The presence of adverse condition for cell survival is signified when ATP is synthesised from intracellular polyphosphate under anaerobic conditions when the ratio of ATP to ADP is low^15,16^. Therefore, PAOs can be distinguished from non-PAOs after incubation for 2 h in phosphate-free medium at pH 4.0 and 3.5 for *L. casei* JCM 1134 and *B. adolescentis* JCM 1275, respectively. Subsequently, the remaining acid-resistant non-PAOs were removed by Percoll density gradient centrifugation based on the fact that such cells are less dense than PAOs (due to stored phosphate). This is the first application of density gradient centrifugation to separate PAOs from non-PAOs on the basis of intracellular phosphate density.

In the next step, a petri-plate method was adopted wherein the TBO dye was added to the growth agar medium with and without additional phosphate, which led to colour development in the grown colonies. The successful selection of the elite PAOs was based on semi-quantitative scores, calculated from the size of the coloured zones formed in the colonies. Elite PAOs of *L. casei* JCM 1134 (L11 isolate) and *B. adolescentis* JCM 1275 (B8 isolate) were isolated successfully on semi-quantitative TBO agar. TBO is a phosphate binding dye and creates a stained area that correlates well with the phosphate accumulation efficiency of the grown colonies. Taken together, our data suggest that the screening of PAOs and their semi-quantitative assessment of phosphate accumulation capabilities can be successfully performed using novel TBO agar strategy in a single step. Unlike other methods, this approach enables direct isolation with the re-cultivation of selected colonies as well as semi-quantitative determination of their phosphate accumulation potential. Furthermore, TBO is safe, metabolically degradable, and requires only general handling.

A few attempts have been made to reduce uremic toxins and treat CKD with probiotics. For example, oral administration of *Lactobacillus acidophilus* for 1–6 months reduced the serum levels of dimethylamine and nitrosodimethylamine, both of which are potent uremic toxins^17^. *Oxalobacter formigenes* was also used to remove high levels of oxalates in patients with urolithiasis^18^. These probiotics may express urease, which can form NH_3_ or NH_4_OH and thus degrade epithelial tight junctions, enable leakage of liposaccharides into the bloodstream, or favour the absorption of uremic toxins and endotoxins^19^.

On the other hand, specific strains are regarded as good PAOs. For example, *Halobacterium salinarium*, *Halorubrum distributum*, and *Brevibacterium antiquum* can store up to 9.5%, 10%, and 13% (w/w) polyphosphate, respectively; in other words, they can accumulate 90%, 90%, and 70% (w/v) phosphate from media containing 8–11 mM orthophosphate^20^. *Brevibacterium casei*, *Acetobacter xylinum*, and *Cryptococcus humicola* were also reported to accumulate 95%, 86%, and 55% (w/v) phosphate from media containing 5 mM KH_2_PO_4_, 5 mM MgSO_4_, 30 mM glucose, and 5 g/L Difco amino acids^21–24^. Similarly, *Saccharomyces cerevisiae*, *Brevibacterium casei*, and *Acetobacter xylinum* can accumulate 0.2, 0.03, and 0.047 [mg-phosphate/mg-dry cell], respectively^23^. Contrary to our results, these strains typically stored more phosphate when supplemented with amino acids. However, *Kuraishia capsulata* and *Saccharomyces cerevisiae* accumulated up to 14% and 70% (w/v) polyphosphate when starved of nitrogen^23^. Collectively, these reports implied that the ability of a microbe to store phosphate and polyphosphate depends to a large extent on its genome and environment.

## Conclusion

In summary, our study successfully isolated elite PAOs via a novel approach and highlights certain points. Firstly, appropriate enrichment method was applied to induce the phosphate accumulation capability of PAOs that could utilize the stored phosphate as a survival strategy at low pH, followed by removal of undesired organisms based on the stored phosphate density. Secondly, the novel semiquantitative TBO assay, which is cheap and safe, can be applied to explore and compare the phosphate accumulation capabilities of potential microbes with required standardization. Thirdly, the current screening method is not species-specific, as relevant metabolic pathways are directly targeted, and hence can be extended or customised based on other requirements for evaluating the phosphate accumulation of other potential microbes. This new strategy of screening and isolating desired microbes is a distinct and efficient way of addressing persistent issues in this field. In this study, strains L11 and B8 emerged as promising biological phosphate accumulators, which may prevent CKD-associated hyperphosphatemia in future. In addition, these PAOs can be delivered as functional food (not medicines), which may prevent CKD at early stages and enhance quality of life.

## Materials and Methods

### Chemicals and equipment

Reagents included De Man Rogosa and Sharpe (MRS) media (Becton Dickinson Difco, U.S.A.), hipolypeptone (Nihon Seiyaku, Japan), beef extract (MP Biomedicals, LLC, France), yeast extract (Becton Dickinson Difco), TBO (Waldeck, Munster), and Percoll (GE Healthcare Life Sciences, Japan). Glucose, Tween 80, K_2_HPO_4_, sodium ascorbate, L-cysteine-HCl, NaNO_3_, MgSO_4_, KH_2_PO_4_, NaH_2_PO_4_, NaCl, and 4′,6-diamidino-2-phenylindole (DAPI) were procured from Fujifilm Wako Pure Chemical Corporation, Japan. Data were collected using a spectrophotometer (UV-1200, Shimadzu, Japan) and a fluorescence microscope DMRXA/RD (Leica Microsystems, Germany). Cultures were prepared using fixed-type (model of rotor: BN 4–6) (H-201FR, Kokusan, Japan) and swing-type (model of rotor: RF 110) centrifuges (H-500FR, Kokusan). Deionised and doubly distilled water was used in all experiments.

### Subculture and maintenance of strains

*L. casei* JCM 1134 and *B. adolescentis* JCM 1275 were inoculated from frozen glycerol stocks to 200 mL Erlenmeyer flasks containing 100 mL sterilised MRS medium and B medium (in w/v%): hi-polypeptone 1, beef extract 0.5, yeast extract 0.5,  glucose 1,  Tween 80 0.1, K_2_HPO_4_ 0.3,  filter-sterilised sodium ascorbate 1, and L-cysteine HCl 0.05, respectively. Cultures were grown for 24 h at 37 °C and sub-cultured for further studies.

### Growth media

*L. casei* JCM 1134 and *B. adolescentis* JCM 1275 were sub-cultured, respectively, in MRS medium supplemented with (in w/v%): K_2_HPO_4_ 0.05,  casamino acids 2, filter-sterilized putrescine 0.17,  and spermidine 0.28; and in modified B medium containing (in w/v%): casamino acids 2, Na-ascorbate 1,  L-cysteine HCl 0.05,  beef extract 0.5, yeast extract 0.5, glucose 1, K_2_HPO_4_ 0.05, filter-sterilized putrescine 0.17, and spermidine 0.28. Cultures were incubated at 37 °C for 24 h.

### Phosphate accumulation media

According to literature, cultures starved of carbon and complex nitrogen sources are depleted of electron acceptors in glycogen utilisation pathways that drive the cells to accumulate phosphate as polyphosphate, an alternative electron acceptor^25^. Further, phosphate accumulation can be enhanced by incorporating polyamines in the media^25^. In this study, the phosphate accumulation media was designed for the enrichment of phosphate accumulation metabolism to induce phosphate uptake by selecting salts from MRS media (as studied microbes were found to be successfully grown on MRS media) and supplementing polyamines. Optimization of phosphate accumulation media was carried out using a one factor at a time approach in which different concentrations of various phosphate salts, nitrogen salts, and salts of trace elements were evaluated (see Supplementary Fig. S2 and Table S1). In the optimised phosphate accumulation media, NO_3_^−^, Na^+^, Mg^2+^, SO_4_^2−^, and K^+^ ions were incorporated to enhance the phosphate accumulation capability that matched well with the published studies^8,26,27^ and the concentrations of salts were optimised without compromising the optimum osmolarity. Active cultures at O.D._680_ = 0.3 were inoculated into optimised phosphate-accumulation medium containing (in w/v%): NaNO_3_ 0.8, MgSO_4_ 0.03, KH_2_PO_4_ 0.332, Na_2_HPO_4_ 1.418, filter-sterilised putrescine 0.17, and spermidine 0.28; and grown at 37 °C and pH 6.0 for 54 h. Samples were collected after every 6 h and assessed for optical density at 680 nm, pH, DAPI staining, CFU/mL and phosphate content using the ascorbic acid method.

### Isolation of elite PAOs

#### First step: selection of pH

After phosphate accumulation from phosphate accumulation medium, cultures were incubated at 37 °C for 7 h in phosphate-free media (phosphate accumulation medium with no phosphate source) at pH 3.5, 4.0, 5.0, 6.0, 7.0, and 8.0. Samples were collected every hour and analysed for O.D._680_ as the growth parameter. In addition, collected samples of *L. casei* JCM 1134 and *B. adolescentis* JCM 1275 at pH 4.0 and 3.5, respectively were evaluated for phosphate content, DAPI staining, and CFU/mL. Further, collected culture samples of *L. casei* JCM 1134 and *B. adolescentis* JCM 1275 at pH 4.0 and 3.5, respectively were incubated in phosphate accumulation media for 18 h at 37 °C to evaluate the enhancement of phosphate accumulation capabilities.

#### Second step: percoll centrifugation

Cultures from phosphate accumulation medium (section: selection of pH) were separated by centrifugation at 1,915 × *g* for 10 min at 25 °C and washed twice with physiological saline. Then, cell pellets of *L. casei* JCM 1134 and *B. adolescentis* JCM 1275 were resuspended in 95% and 70% (v/v) Percoll, respectively, with NaCl of suitable osmolarity and centrifuged at 1,100 × *g* for 10 min at 25 °C using swing-type centrifuge. The conditions of centrifugation and the percentage of Percoll were optimised to obtain PAOs over non-PAOs, based on the difference in the density of stored phosphate inside cells, which was confirmed by DAPI staining. After centrifugation, the denser parts, with high yellow fluorescence in DAPI staining, were collected and considered as PAO strains. The collected samples were cultured in growth media and assayed for phosphate accumulation capability after incubation in phosphate accumulation media for 18 h at 37 °C.

#### Third step: TBO agar assay

After Percoll gradient centrifugation, 15 isolates of PAO strains of *L. casei* JCM 1134 and 10 isolates of PAO strains of *B. adolescentis* JCM 1275 were picked from their respective growth agar-media and sub-cultured in MRS and B media, respectively, and stored in 20% (v/v) glycerol at −80 °C. To obtain elite PAO strains, obtained PAOs were diluted suitably in physiological saline and plated on the following media: for *L. casei* JCM 1134, with and without 2% w/v K_2_HPO_4_-supplemented MRS (without and with 0.0025% w/v TBO), and for *B. adolescentis* JCM 1275, with and without 2% w/v K_2_HPO_4_-supplemented B agar (without and with 0.0025% w/v TBO). After 24 h incubation at 37 °C, the colonies were analysed under a light microscope.

### Data processing of third step: Semi-quantitative estimation of accumulated phosphate

Phosphate content was estimated semi-quantitatively by analysing the snapshots (captured using a light microscope) of colony size on Microsoft PowerPoint 2016 version 15.27. Briefly, snapshots of colonies were pasted on PowerPoint slides and colony size was estimated (with or without background) using the Shape Size tool (the vertical and horizontal diameter). Background was deleted as required using the ‘Remove Background’ tool. To avoid ambiguity, phosphate accumulation was normalised to colony size using the following equation:

1 $$P=(\frac{a}{b})-(\frac{c}{d})$$

where P is phosphate accumulation score, a is stained area in phosphate-rich media (cm), b is stained area in phosphate-deficient media (cm), c is colony size in phosphate-rich media (cm), and d is colony size in phosphate-deficient media (cm). This is a semi-quantitative method based on relative phosphate accumulation scores for relating the phosphate accumulation capabilities of the isolated PAO strains, which were later compared with the results of the quantitative ascorbic acid assay by incubating active cultures in phosphate accumulation media for 24 h at 37 °C and also assayed for phosphate accumulation using DAPI staining method.

### Analytical techniques

#### Cell growth

Samples were diluted as required and optical density was assayed at 680 nm using a spectrophotometer (UV-1200, Shimadzu). CFU/mL was estimated by spread-plating the appropriately diluted samples of *L. casei* JCM 1134 and *B. adolescentis* JCM 1275 on MRS and B medium agar (2% w/v), respectively.

#### Measurement of phosphate using DAPI and ascorbic acid

Broth samples (245 µL) were stained with 12.5 µL DAPI (1,000 μg/mL in 0.025 M Tris-HCl), mounted on glass slides and analysed using UV-fluorescence microscopy^28^. Phosphate content was also assessed using the ascorbic acid method according to published methods^29^, with some modifications. Briefly, the collected samples were appropriately diluted and hydrolysed for 30 min at 121 °C using peroxodisulphuric acid potassium (0.25 g) and 5 N sulphuric acid (0.54 mL). After hydrolysis, the samples were filtered using 0.2 µm filters, and assessed for phosphoric acid by mixing 4.2 mL filtered samples with 0.8 mL of a 10:3:6:1 mixture of 5 N sulphuric acid: K_2_(SbO)_2_C_8_H_4_O_10_·3H_2_O, 0.1372 g/100 mL: (NH_4_)_6_Mo_7_O_24_·4H_2_O, 4.0 g/100 mL: ascorbic acid, 1.32 g/75 mL + formic acid, 0.6 g in 0.4954 mL + EDTA, 25 mg. Phosphoric acid was used as the standard. Reactions were assayed at 880 nm after 10 min incubation at room temperature.

### Statistical analysis

All the experiments were performed in triplicate (n = 3). An analysis of variance (ANOVA) was performed for the multiple comparisons between data sets using Microsoft Excel 2016 (version 15.26) to determine the significant differences (*p* < 0.05). Error bars (standard error of mean) are included for all the numerical data.

## Supplementary information

Dataset Figure S1, Figure S2, Table S1

## Data Availability

All data generated or analysed during this study are included in this article.
