# Supplementary material for: A High Throughput Isolation Method for Phosphate-Accumulating Organisms
Source: Sci Rep. 2019 Dec 2;9:18083. doi: 10.1038/s41598-019-53429-2 (PMC6888830; doi:10.1038/s41598-019-53429-2)
Supplement: Supplementary file 1 — Dataset Figure S1, Figure S2, Table S1 [file 41598_2019_53429_MOESM1_ESM.pdf]

A High Throughput Isolation Method for Phosphate-accumulating Organisms

Ajeeta Anand,[a,#] Hideki Aoyagi[b]*

LIST OF SUPPLEMENTARY FIGURE LEGENDS AND TABLES

SUPPLEMENTARY FIGURE S1 Enhancement in phosphate accumulation capability during various stages of PAO isolation.

SUPPLEMENTARY FIGURE S2 One factor at a time approach was applied to optimise the phosphate accumulation medium for the maximum accumulation of phosphate from the medium (*L. casei* JCM 1134: white bar and solid black line, *B. adolescentis* JCM 1275: grey bar and dotted line) by cells of optical density 0.3 at 680 nm. (A) Bar graph→ phosphate salts were evaluated as suitable phosphate source,; line graph→ ratio of two best phosphate salts (KH_2PO_4 and Na_2HPO_4) were analysed, where square, triangle, and circle represent ratios 1:1, 1.5:0.5, and 0.5:1.5 (w/v), respectively. (B) Bar graph→ nitrogen salts were evaluated as suitable nitrogen source; line graph→ different concentrations of sodium nitrate were analysed, where black triangle, black square, white circle, white triangle, and black circle represent ratios of 1.4, 1.2, 1.0, 0.8 and 0.6 (w/v), respectively. (C) Bar graph→ salts of trace elements were evaluated as suitable cofactor; line graph→ different concentrations of magnesium sulphate were analysed, where black circle, black triangle, black square, white circle, and white triangle represent ratios of 0.09, 0.07, 0.05, 0.03 and 0.01 (w/v), respectively. Experiments were conducted in triplicates and standard errors were found to be less than 3% of the observed values.

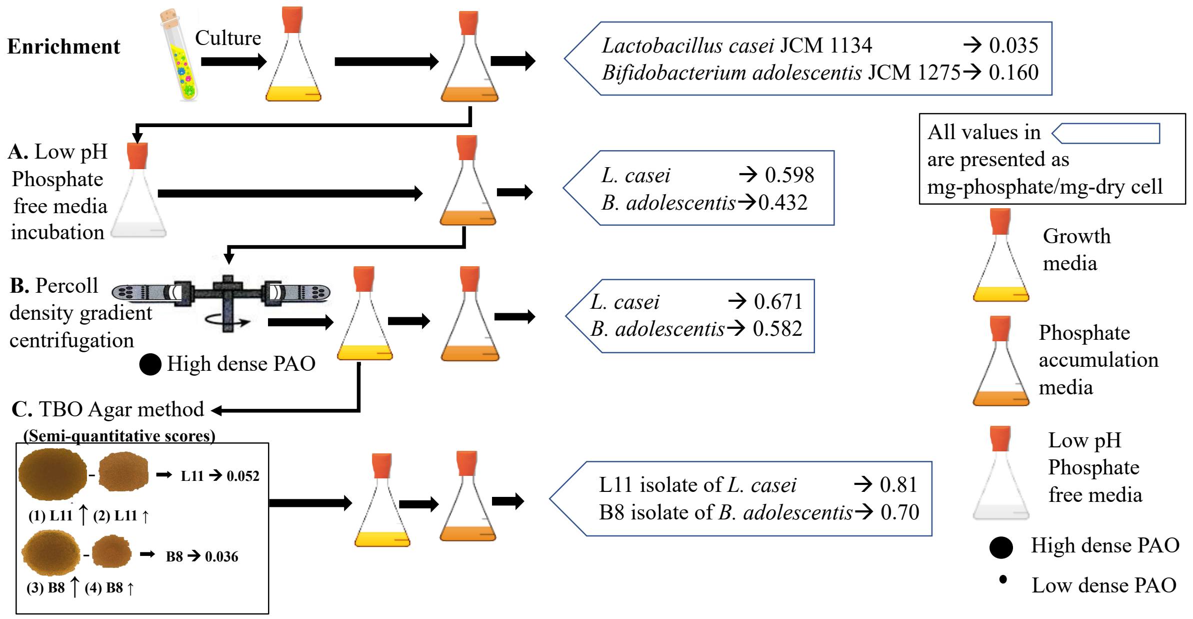

SUPPLEMENTARY FIGURE S1 Enhancement in phosphate accumulation capability during various stages of PAO isolation.

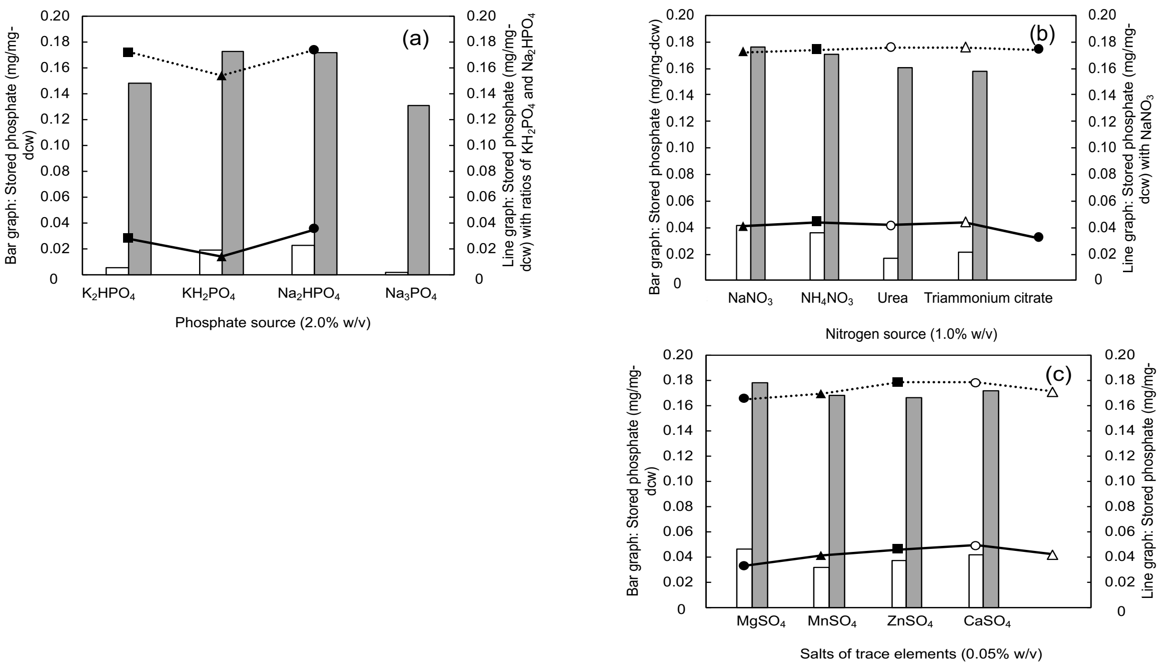

SUPPLEMENTARY FIGURE S2 One factor at a time approach was applied to optimise the phosphate accumulation medium for the maximum accumulation of phosphate from the medium (*L. casei* JCM 1134: white bar and solid black line, *B. adolescentis* JCM 1275: grey bar and dotted line) by cells of optical density 0.3 at 680 nm. (A) Bar graph→ phosphate salts were evaluated as suitable phosphate source,; line graph→ ratio of two best phosphate salts (KH_2PO_4 and Na_2HPO_4) were analysed, where square, triangle, and circle represent ratios 1:1, 1.5:0.5, and 0.5:1.5 (w/v), respectively. (B) Bar graph→ nitrogen salts were evaluated as suitable nitrogen source; line graph→ different concentrations of sodium nitrate were analysed, where black triangle, black square, white circle, white triangle, and black circle represent ratios of 1.4, 1.2, 1.0, 0.8 and 0.6 (w/v), respectively. (C) Bar graph→ salts of trace elements were evaluated as suitable cofactor; line graph→ different concentrations of magnesium sulphate were analysed, where black circle, black triangle, black square, white circle, and white triangle represent ratios of 0.09, 0.07, 0.05, 0.03 and 0.01 (w/v), respectively. Experiments were conducted in triplicates and standard errors were found to be less than 3% of the observed values.

SUPPLEMENTARY TABLE S1 Composition of pre-optimised phosphate accumulation media.

**Composition**	**% (w/v)**
K_2HPO_4	0.2
$MgSO_4$	0.02
$MnSO_4$	0.005
NH_4NO_3	1.0
pH	6.0
Temperature	37°C
Time	36 h

Optical density at 680 nm for (*L. casei* JCM 1134 and *B. adolescentis* JCM 1275) cells was 0.3, and filter-sterilised putrescine (0.17 % w/v), and spermidine (0.28 % w/v) were added.
